# Supplementary material for: Impact of integrating family planning with maternal and child health on uptake of contraception: A quasi-experimental study in rural, Sindh, Pakistan
Source: PLOS Glob Public Health. 2025 Jul 8;5(7):e0004872. doi: 10.1371/journal.pgph.0004872 (PMC12237021; doi:10.1371/journal.pgph.0004872)
Supplement: S1 Table — (DOCX) [file pgph.0004872.s001.docx]

### **S1_Table : Selection of Control District using Propensity Score Matching (PSM) from 23 rural districts of Sindh**

| **SN** | **Districts** | **UMN** | **CPR** | **mCPR** | **HDI** | **FIMM** | **DSBA** | **ANC** | **Propensity Score(PSM)** |
| --- | --- | --- | --- | --- | --- | --- | --- | --- | --- |
| 1 | **Badin** | 17.9 | 28.1 | 28.1 | 41 | 45.6 | 61.9 | 82 | **0.36** |
| 2 | Sanghar | 21.7 | 24.9 | 24.9 | 49 | 59.4 | 59.4 | 76 | 0.19 |
| 3 | **Matiari** | 25.8 | 32.4 | 30.6 | 57 | 68 | 65.5 | 85.7 | **0.31** |
| 4 | Qamber Shahdadqot | 19.3 | 18.4 | 18.2 | 46 | 22.6 | 52.4 | 70.3 | 0.06 |
| 5 | Kashmore | 26.1 | 16 | 15.7 | 47 | 9.7 | 35.9 | 63.5 | 0.12 |
| 6 | Jacobabad | 22.8 | 19.9 | 16.9 | 44 | 18 | 51.9 | 59.5 | 0.00 |
| 7 | Larkana | 20.6 | 26.7 | 24.3 | 62 | 41 | 55.8 | 70.3 | 0.04 |
| 8 | Shikarpur | 26.9 | 11.3 | 10.8 | 52 | 32 | 47.3 | 72.6 | 0.02 |
| 9 | Ghotki | 24.5 | 22.2 | 21.4 | 51 | 19.6 | 43.1 | 55.4 | 0.09 |
| 10 | Sukkur | 20.9 | 27.2 | 26.9 | 66 | 27.9 | 72.8 | 69.4 | 0.14 |
| 11 | Khairpur | 22.4 | 19.7 | 18.7 | 56 | 29.8 | 55.5 | 90.8 | 0.17 |
| 12 | Naushahro Feroz | 21.7 | 20.7 | 18.5 | 67 | 62.5 | 53.4 | 75.8 | 0.01 |
| 13 | Shaheed Benazirabad | 22.8 | 23.9 | 22 | 57 | 36.4 | 63.7 | 76.8 | 0.04 |
| 14 | Dadu | 21.8 | 19.7 | 18.8 | 63 | 18.9 | 50.2 | 74.5 | 0.10 |
| 15 | Jamshoro | 20.7 | 21.9 | 21.4 | 57 | 68.5 | 57.2 | 75.3 | 0.06 |
| 16 | Hyderabad | 17.5 | 33.3 | 28.9 | 72 | 61.3 | 90.6 | 95 | 0.01 |
| 17 | Tando Allahyar | 21.4 | 28.8 | 26.1 | 53 | 63.8 | 67.4 | 91.7 | 0.09 |
| 18 | Tando Muhammad Khan | 21.2 | 28.5 | 26.4 | 38 | 32.1 | 71.8 | 79.9 | 0.08 |
| 19 | Sujawal | 26.2 | 15.9 | 15.3 | 33 | 40.2 | 52.6 | 79.3 | 0.06 |
| 20 | Thatta | 22.6 | 19.7 | 15.9 | 38 | 37.4 | 59.4 | 74.1 | 0.00 |
| 21 | Mirpurkhas | 23.9 | 24.5 | 21.9 | 43 | 38.1 | 51.8 | 61.9 | 0.02 |
| 22 | Umerkot | 22.4 | 19.2 | 19 | 32 | 60.1 | 37.3 | 55.8 | 0.04 |
| 23 | Tharparkar | 30.9 | 12.1 | 11.7 | 23 | 31.3 | 20.7 | 30.6 | 0.01 |

Unmet Need (UMN), Contraceptive Prevalence Rate (CPR), Modern Contraceptive Prevalence Rate (mCPR), Human Development Index (HDI), Female literacy and Maternal Mortality (FIMM), Deliveries by Skilled Birth Attendants (DSBA), and Antenatal Care (ANC) coverage
