# Supplementary material for: Impact of integrating family planning with maternal and child health on uptake of contraception: A quasi-experimental study in rural, Sindh, Pakistan
Source: PLOS Glob Public Health. 2025 Jul 8;5(7):e0004872. doi: 10.1371/journal.pgph.0004872 (PMC12237021; doi:10.1371/journal.pgph.0004872)
Supplement: Attachment — Submitted filename: Feedback on Manuscript PGPH-D-24-01352.pdf [file pgph.0004872.s004.pdf]

Kindly provide scientific evidence or rationale on the following comments related to the design and analysis before the manuscript can be considered for publication:

Title: " Rural Pakistan " is confusing. How come only the catchments (the design suggests that the intervention was implemented at the catchment level, not at the district level) of two districts of Sindh province can represent rural Pakistan?

Selection of intervention and control – what are the criteria of the selection of intervention and control districts, especially when the below indicators do not match/gross mismatch based on the MICS 2018-19 data from Sindh, Pakistan

- Key HH characteristics do not match (gross mismatch) between intervention and control, like electricity, energy used for cooking, the percentage of HH assets, the main material for HH flooring/roofing/walls, high women's illiteracy, women's exposure to mass media, etc.
- Pre-mature births: 30.6% in Matiari and 7.2% in Badin
- Adolescent birth rate and total fertility rate: Badin 60.5 and 4.7 while Matiari 40.5 and 3.5
- Use of any modern method of contraception among currently married women is 33.6% for Badin and 25.4% for Matiari
- Unmet need for contraception is 18.8% for Badin while 29.1% for Matiari
- FP counselling is 52.5% and 30.6% in Matiari
- Antenatal coverage for Badin is 78%, while for Matiari is 93.5%
- Skilled birth is 66.4% in Badin and 80.1% in Matiari
- In terms of wealth quintiles, Badin is one of the poorest districts (a total of 80% are poor (i.e. fall under the poorest [62.2%] and poor/second quintile [17.9%] of Sindh). In Matiari, a total of 55.7% are poor (the poorest are 25.3% and poor/second quintile 30.4%).
- Regarding the wealth disparity, the results also indicate that “difference in wealth
- quantiles between the intervention and control groups highlight existing disparities that may
- impact study findings”. Then why did researchers select two districts with quite different health and SES indicators?

Data collection: I am concerned about the quality and integrity of the data collected and the of data collection process; what the process of identifying the sample clients from baseline to endline, for example:

- The baseline survey was implemented at the population level from 1 October to 31 December 2020, followed by a follow-up household survey from 1 October to 31 December 2022.
- The baseline HH data was collected during the COVID-19 outbreak and lockdowns.
- Likewise, the endline HH data was collected during Pakistan's severe flash floods/monsoon rains. Such events push the affected families out of their areas/HH for safety, shelter, food and health care.
- So, how did the researchers ensure that the baseline and endline respondents' HH were the same??

Intervention: Below is your intervention package. Are you measuring the impact of the overall package, or do you have information on what part of the intervention actually worked and what did not?

1. Integration of FP-At three points
2. Capacity Building-Training of providers
3. Integration at the community level-Revitalizing women support group (WSG), village health committee (VHC) sessions
4. Strengthening supply chain of FP commodities-District health office (DHO)

Analysis and Results: Thank you for conducting the DiD analysis. However, update the tables to present the correct labelling, add "Absolute Difference" and "Net Effect", and then the p-values. Also, put the proper values with decimals, not rounded figures.

In addition, the district-level MICS 2018-19 data shows that the following indicators are already high in the intervention and control districts; how did you see it was your intervention that was able to make this change? Kindly justify as you are reporting an increase in the below indicators, but with the routine trend reported in MICS survey data, these districts are performing well in these indicators:

- Use of any modern method of contraception among currently married women is 33.6% for Badin and 25.4% for Matiari
- Unmet need for contraception is 18.8% for Badin while 29.1% for Matiari
- FP counselling is 52.5% and 30.6% in Matiari
- Antenatal coverage for Badin is 78%, while for Matiari is 93.5%
- Skilled birth is 66.4% in Badin and 80.1% in Matiari
- Results are significant for STM (INJ and condom) and LAM
- Badin reports INJ use at 8.3%, while Matiari reported 9.6% INJ use (MICS)
- Badin reports CONDOM use at 6.4%, while Matiari reported 1.8 % CONDOM use (MICS)
- So, the change in net effect seems like a normal trend
- Also, I do not see any strong evidence of FP-integration with MCH work in the paper based on the limited number of indicators that showed improvement in the catchment areas where the study was implemented.

It seems like the overall presentation and formatting of tables were done in a rush. Kindly look at the formatting of the tables. Also, the manuscript required Native English editor proofing and copy-editing services to improve the language flow, comprehension, and writing.
